# DAG tales: the multiple faces of diacylglycerol—stereochemistry, metabolism, and signaling

**DOI:** 10.1007/s00018-015-1982-3

**Published:** 2015-07-08

**Authors:** Thomas Oliver Eichmann, Achim Lass

**Affiliations:** Institute of Molecular Biosciences, University of Graz, Heinrichstrasse 31/2, 8010 Graz, Austria

**Keywords:** Lipase, Hydrolase, Acyltransferase, Kinase, Insulin

## Abstract

The neutral lipids diacylglycerols (DAGs) are involved in a plethora of metabolic pathways. They function as components of cellular membranes, as building blocks for glycero(phospho)lipids, and as lipid second messengers. Considering their central role in multiple metabolic processes and signaling pathways, cellular DAG levels require a tight regulation to ensure a constant and controlled availability. Interestingly, DAG species are versatile in their chemical structure. Besides the different fatty acid species esterified to the glycerol backbone, DAGs can occur in three different stereo/regioisoforms, each with unique biological properties. Recent scientific advances have revealed that DAG metabolizing enzymes generate and distinguish different DAG isoforms, and that only one DAG isoform holds signaling properties. Herein, we review the current knowledge of DAG stereochemistry and their impact on cellular metabolism and signaling. Further, we describe intracellular DAG turnover and its stereochemistry in a 3-pool model to illustrate the spatial and stereochemical separation and hereby the diversity of cellular DAG metabolism.

## Introduction

For a long time, diacylglycerol (DAG) has been recognized as lipid molecule which exhibits signaling function. More recently, research has unraveled that certain lipid-modifying enzymes discriminate between different stereo/regioisomers of DAG, pinpointing that different DAG isomers may have distinct cellular functions and fates. Thus, the stereochemical nature of DAG isomers by itself is a determinant for its physiological role in distinct cellular compartments and metabolic pathways.

## Stereochemistry of DAG

**Fig. 1:**
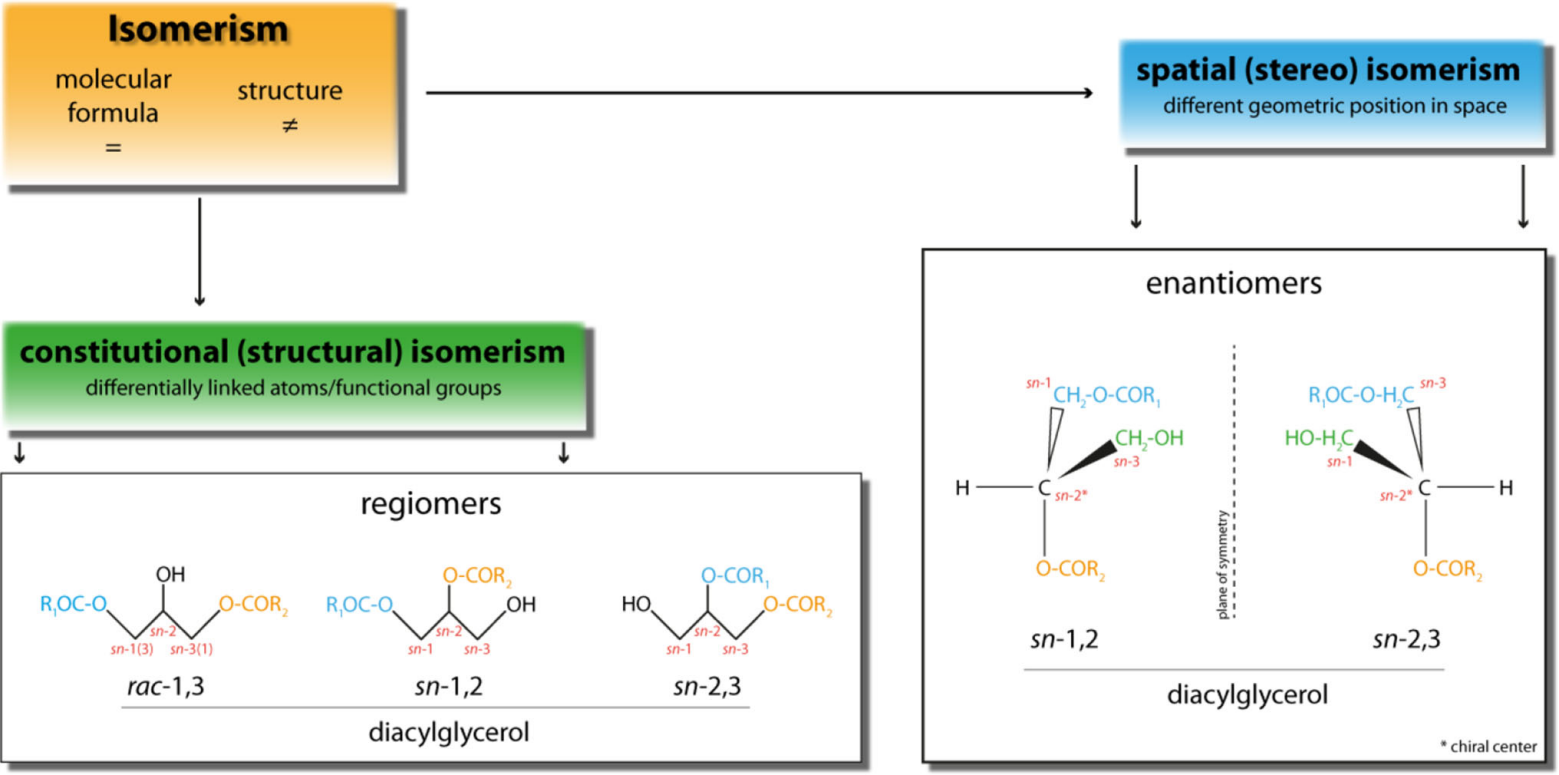
Schematic depiction of the different forms of isomerism of diacylglycerol. Diacylglycerols feature different forms of isomerism and can differ either in constitutional (structural) or spatial (stereo) conformation. For detailed explanation see text

Generally, isomers (from Greek: isos—equal, mèros—part) are molecules sharing identical molecular formulas but differing in their structures. Isomers can be divided into two main groups. On the one hand, there are structural isomers which exhibit differentially linked atoms and functional groups. On the other hand, there are spatial isomers (stereoisomers) which display same linkage of atoms and functional groups but differ in their geometrical position in space. A special group of stereoisomers, named enantiomers, is related by reflection which implies that two enantiomers are not superimposable. Furthermore, enantiomers are characterized by an asymmetric or chiral carbon atom, featured by four different ligands (Fig. 1).

DAGs are generated during a variety of metabolic reactions and have attracted much attention as important signaling molecules. Importantly, DAG represents a lipid class that exhibits different isomeric properties. Triacylglycerol (TAG), one possible metabolic precursor of DAG, contains three fatty acids (FAs) esterified to the trihydric alcohol glycerol. This implicates that TAG provides three possible sites for lipase-dependent hydrolysis, which can result in three different DAG isoforms. The stereospecific numbering (*sn*) designates the conformation of glycerol derivatives, hence the position of the fatty ester at the glycerol backbone (*sn*-1, *sn*-2, *sn*-3; according to IUPAC nomenclature). Accordingly, DAGs generated from the hydrolysis of the phospholipid (PL) headgroup are per definition *sn*-1,2 isomers since *sn*-glycerol-3-phosphate/l-α-glycerophosphate (G3P) is the basic building block of all PLs.

TAG exhibits another important property as lipase substrate, namely prochirality. Prochirality describes the condition that an achiral molecule can be converted into a chiral molecule by a one-step reaction. In case of TAG, the potential achiral carbon atom at *sn*-2 position becomes a chiral center by removal of the attached FA either at *sn*-1 or at *sn*-3 position.

**Fig. 2:**
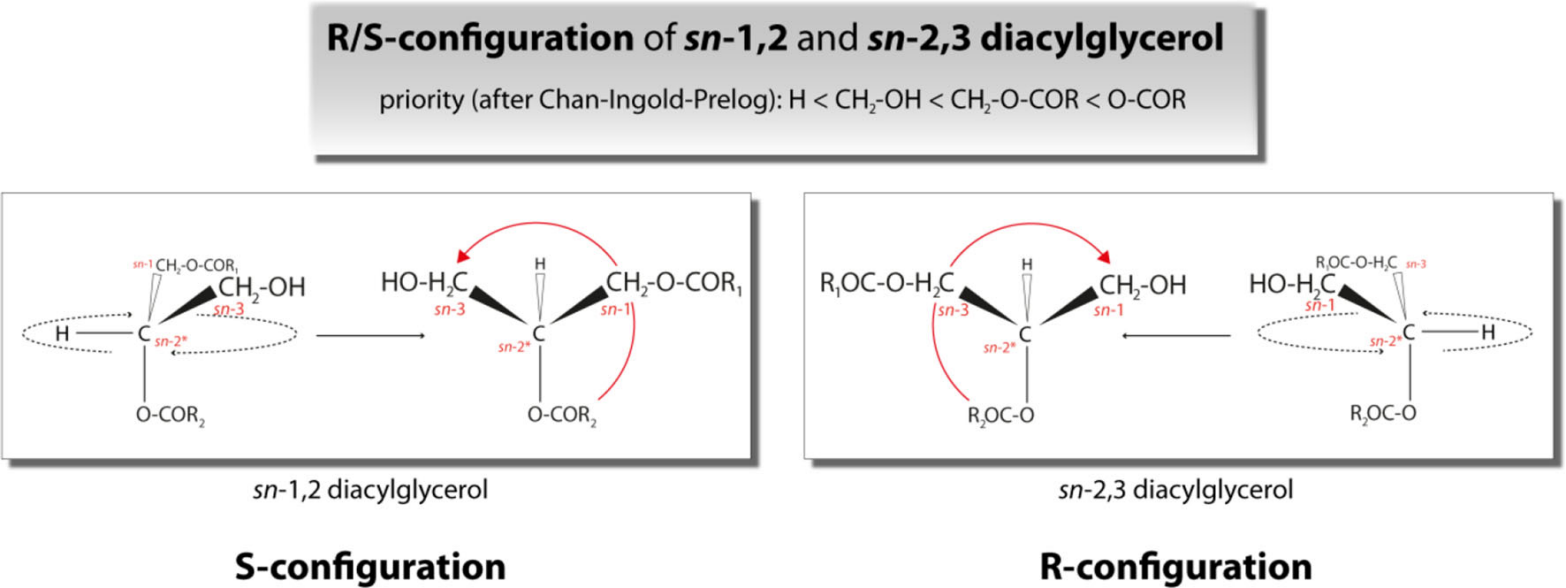
R/S nomenclature of diacylglycerol (DAG) enantiomers according to Cahn–Ingold–Prelog convention. *sn*-1,2 DAG represents the S-configuration whereas *sn*-2,3 DAG represents the R-configuration

Prochiral TAG species exhibit chemically identical but enantiotopic reactive groups (e.g., oleic acid (C18:1) at *sn*-1 and *sn*-3 position). These groups (FAs) can be stereochemically discriminated during lipase-dependent hydrolysis reaction, which results in a chiral DAG product. Lipase-dependent cleavage of the FA esterified at either *sn*-1 or *sn*-3 position of a TAG molecule leads to the generation of a chiral center at the *sn*-2 position and to one of the two DAG enantiomers, *sn*-2,3 or *sn*-1,2 DAG, respectively. These DAG enantiomers face themselves as reflection and are not superimposable (Fig. 1). Importantly, 1,3 DAGs can be achiral or chiral, depending on the fatty acid species esterified to the *sn*-1 and *sn*-3 position. If two identical fatty acid species are esterified to both position then 1,3 DAG is achiral while if fatty esters at the *sn*-1 and *sn*-3 position are dissimilar then 1,3 DAG is chiral. Since in many studies neither fatty ester species nor their respective position on the 1,3 DAG molecule have been determined we refer to 1,3 DAG as racemic/*rac*-1,3 DAG throughout this review to account for unknown stereochemistry. Furthermore, enantiomeric DAGs can be classified in respect to the R/S-configuration nomenclature based on the Cahn–Ingold–Prelog (CIP) system. The CIP system is used to uniquely specify enantiomers. Therefore, priorities are assigned to all groups attached to the chiral center (CIP rules) [1, 2]. Subsequently, the lowest ranked group is set below the image plane and the other groups are counted starting at highest priority substituents. The counted sequence can be either clockwise or counterclockwise and specifies the present molecule as either R-configured (from Latin: *rectus*—right) or S-configured (from Latin: *sinister*—left). In case of DAG, *sn*-1,2 DAG reflects the S-configuration whereas *sn*-2,3 DAG is R-configured (Fig. 2).

Upon hydrolysis of the *sn*-2-bound FA of a TAG molecule, the generated DAG exhibits *rac*-1,3 conformation. In regard to the different region of hydrolysis (*sn*-1/*sn*-3: esters of a primary alcohol; sn-2: ester of a secondary alcohol), *rac*-1,3 DAG is a so-called regiomer (Fig. 1). Thus, lipases can regioselectively differentiate between *sn*-2 and *sn*-1/*sn*-3 position or enantioselectively differentiate between *sn*-1 and *sn*-3 position. Similarly, other DAG metabolizing enzymes, such as acyltransferases may also discriminate between different isoforms of DAGs.

These differences in DAG isomerisms as well as in the selectively of DAG-generating/consuming enzymes are an important issue when DAGs which represent an intersection point between lipid and signaling metabolism are investigated. The selectivity of cellular lipases/acyltransferases as well as the impact of DAG isomerism on cellular metabolism is important to fully understand cellular functions of different DAG molecules.

## Intracellular formation of DAG

**Fig. 3:**
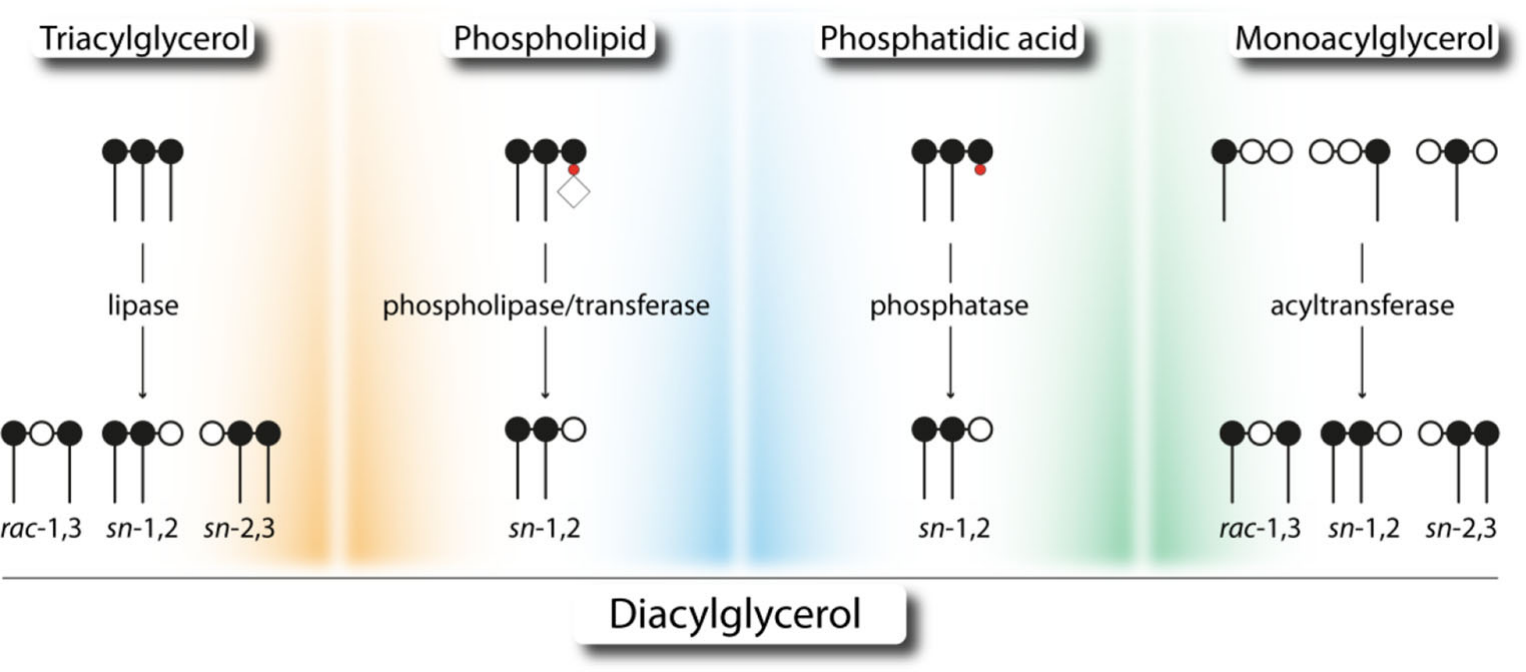
Catabolic and anabolic reactions leading to the formation of diacylglycerol. Different stereo/regioisomers of diacylglycerols are generated by the hydrolysis of either triacylglycerol (lipase) or phospholipids (phospholipase), and are product of sphingomyelin synthesis (transferase reaction). Furthermore, diacylglycerol is the product of the dephosphorylation of phosphatidic acid (phosphatase) and of the esterification of monoacylglycerol by acyltransferases. Carbon atoms of the glycerol backbone are depicted as *filled* (esterified) or *open* (unesterified) *circles*. Phosphate group, phosphate head group, and fatty acids are depicted as *red circle*, *open rhomb*, and *dash*, respectively

Intracellularly, several reactions contribute to the generation of DAG and are located at different subcellular compartments, including the endoplasmic reticulum (ER), the Golgi network, lipid droplets (LDs), and the plasma membrane. Therefore, either TAG, stored in cytoplasmic or ER-associated LDs or PLs, which are building blocks of cellular membranes, can act as source for lipase-, or acyltransferase-dependent generation of DAG. Additionally, DAGs are also an intermediate during de novo synthesis of TAGs either generated by acyltransferases or by phosphohydrolases. The stereo/regioselectivity of involved enzymes and the isomerism of formed DAGs are largely unknown but might be crucial for subsequent cellular reactions. The following sections describe known biochemical characteristics and stereo/regiochemical properties of enzymes involved in the formation of DAGs (Fig. 3).

### Formation of DAG by neutral triglyceride lipases

Within most cell types, TAG turnover is crucial to balance energy storage and utilization. In whole body energy metabolism of higher organisms, this function is mainly achieved by specialized cells, named adipocytes and hepatocytes, constituents of the white adipose tissue (WAT) and the liver, respectively. In adipocytes, excessive energy is stored in form of TAG in cytoplasmic LDs. Interestingly, adipocytes usually harbor one giant LD with a size range of about 100 µm, while non-adipocytes exhibit multiple LDs with a diameter of around 1 µm. Irrespectively thereof, all LDs share basically the same architecture. The core is strictly assembled by hydrophobic lipid esters, like TAG and cholesteryl esters (CEs), and the surface is formed by a PL monolayer [3]. This monolayer harbors a variety of anchored or embedded proteins and serves as an amphipathic shield against the aqueous milieu present within the cell [4]. The most important function of adipocyte LDs is the storage and the lipase-dependent release of energy metabolites, primarily in form of FAs. This tightly regulated process of TAG degradation, known as lipolysis, is initiated by TAG lipases that generate DAGs and FAs.

Hepatocytes are a crucial intersection of the energy metabolism by receiving, remodeling, and distributing lipids from and to the systemic circulation. Hepatocytes incorporate circulating FAs into TAGs which are stored either in cytoplasmic LDs or used for very low density lipoprotein synthesis within the lumen of the endoplasmic reticulum (ER) [5]. Both, the cytoplasmic and the luminal TAG stores are accessible for different lipases.

#### Adipose triglyceride lipase generates *rac*-1,3 DAG at the LD

In 2004, adipose triglyceride lipase (ATGL) was independently identified by three laboratories. The former denoted transport secretion protein 2.2 was renamed as ATGL/patatin-like phospholipase domain containing 2 (PNPLA2) [6], desnutrin [7], and calcium-independent phospholipase A2ζ (iPLA2ζ) [8].

ATGL is one of nine PNPLA family members found in humans (PNPLA1–9) [9]. The PNPLA protein family is named after the patatin-domain which was first identified in hydrolases of the potato plant and denominated after the most abundant protein of the potato tuber, patatin. Lipid hydrolases, containing this domain, catalyze the non-selective hydrolysis of a variety of lipids, including PLs, glycolipids, DAGs, and monoacylglycerols (MAGs) [10, 11]. Orthologs of ATGL exist in almost every eukaryotic species including invertebrates, fungi, and plants.

ATGL localizes to cytosolic LDs [12] and exhibits highest hydrolytic activity for TAGs, much less for retinyl esters (REs) and no detectable activity for other neutral lipids such as DAGs, MAGs, and CEs [6, 13]. Additionally, phospholipase A2 (PLA2) activity as well as DAG transacylase activity have been also reported but the physiological relevance of these activities has not been established [8, 14].

In mice, ATGL mRNA is expressed in all examined tissues. Highest expression is observed in WAT and brown adipose tissue (BAT) and is manifold increased upon fasting. Lower expression levels are detectable in skeletal muscle, cardiac muscle, liver, and testis [6, 7, 15, 16]. Interestingly, besides nutritional regulation at the mRNA level [6, 7, 17, 18] ATGL’s activity is highly regulated at the post-transcriptional level by the interaction with an activator and inhibitor protein [19, 20]. Binding of molar amounts of comparative gene identification-58 (CGI-58; or α/β hydrolase fold domain containing protein 5, ABHD5) stimulates ATGL’s activity up to 20-fold [19, 21]. In contrast, binding of molar amounts of G_0_/G_1_ switch gene 2 (G0S2) to ATGL complete abolishes ATGL’s hydrolase activity [20, 21]. Interestingly, CGI-58 and G0S2 regulate ATGL in a non-competitive manner, although the molecular mechanism behind CGI-58- and G0S2-dependent regulation of ATGL’s activity is unknown.

The key role of ATGL in the degradation of TAGs is evident by the phenotype of ATGL-deficient mice. ATGL knockout mice exhibit enlarged adipose tissues. TAG accumulation in LDs is observable in all tissues, reaching up to tenfold increase in TAG content [22]. These observations consolidate ATGL as the rate limiting lipase in cytoplasmic TAG mobilization of adipose and non-adipose tissues.

Studies on substrate and stereospecificity revealed that ATGL hydrolyzes long-chain fatty acids of various length and saturation. Importantly, ATGL is unique among mammalian lipases by hydrolyzing TAGs selectively at *sn*-2 position. In the absence of its co-activator protein CGI-58, ATGL generates *rac*-1,3 DAGs. Interestingly, upon co-activation by CGI-58, ATGL expands its regioselectivity to the *sn*-1 position, generating additionally *sn*-2,3 DAGs. Notably, ATGL does not hydrolyze ester bonds at the *sn*-3 position of TAGs, hence does not generate detectable amounts of *sn*-1,2 DAGs [23].

#### Hormone-sensitive lipase hydrolyzes lipolysis-derived DAGs at the *sn*-3 position

Hormone-sensitive lipase (HSL) was initially identified following up the observation that WAT lipolysis is strongly inducible by hormones [24, 25]. HSL is expressed in many tissues; protein as well as mRNA expression of HSL are highest in WAT and BAT. Expression levels are barely increased upon fasting suggesting that its activity is largely regulated by post-translational events [26, 27]. The COOH terminus of HSL harbors an α/β hydrolase fold domain, containing an active site serine (Ser^423^) as part of a catalytic triad (Asp^703^, His^733^), responsible for hydrolytic activity [28–31]. The lipid-binding site as well as the site responsible for protein dimerization is located at the NH_2_-terminal region of HSL [32].

HSL-dependent activity is strongly regulated by hormones. A variety of phosphorylation events controls HSL activity by affecting both intracellular localization and protein–protein interactions. The major positive stimulus is caused by catecholamines which bind to β-adrenergic receptors during periods of nutritional deprivation (fasting). The contrary nutritional condition (feeding) inhibits HSL activity via insulin [33–39]. Even though phosphorylation events of HSL are crucial, they cause only moderate changes in enzymatic activity (~twofold). The more important factor in HSL activation is the translocation of the enzyme from the cytosol to the LD. In adipocytes, LDs are shielded by perilipin-1 which surrounds LDs, forming a barrier between lipases and respective substrates and prevents effective lipolysis [40, 41]. Upon β-adrenergic stimulation perilipin-1 is phosphorylated [42–45] which causes HSL to bind to perilipin-1. Thereby, HSL translocates to the LD surface and deploys full activity [46, 47].

The long-standing dogma that HSL acts as pacemaker of lipolysis, thereby hydrolyzing TAGs and DAGs, was disproven when mice, carrying a global deletion of HSL, showed no signs of obesity on a high-fat diet but exhibited normal bodyweight and even reduced fat mass [48, 49]. Decreased adipose mass was partially explained by reduced FA esterification, counteracting decreased lipolytic activity [50]. Noteworthy, HSL-deficient adipocytes remain catecholamine-inducible and exhibit increased FA release indicating that an additional TAG lipase must exist [48, 51, 52]. However, the most intriguing finding in HSL knockout mice was the drastic accumulation of DAG in several tissues suggesting that HSL is responsible for DAG hydrolysis [51].

Remarkably, HSL exhibits a uniquely broad substrate spectrum which includes TAGs, DAGs, MAGs, CEs, REs, and the artificial water-soluble esters of *para*-nitrophenol [25, 53–55]. However, in vitro studies demonstrated that the specific activity of HSL is highest against DAG which exceeds those against TAG and MAG around tenfold [53, 56]. Earlier studies reported specificity of HSL for *sn*-1/(3) DAGs [56], whereas most recently HSL was identified to be *sn*-3 specific [57]. Concerning substrate specificity, HSL exhibits preference for polyunsaturated FAs (*n-*3/*n-*6) [58].

Both ATGL and HSL together are responsible for more than 90 % of lipolytic (TAG hydrolase) activity as determined in cultured adipocytes and murine WAT [59]. This suggests that other lipases may contribute to a minor extent to TAG breakdown in adipose tissue. Several other enzymes which are known to catabolize TAG breakdown are discussed below.

#### Triacylglycerol hydrolase/carboxylesterase-3 hydrolyzes short-chain TAGs at the ER

Triacylglycerol hydrolase/carboxylesterase-3 (TGH/Ces3) was initially purified from porcine liver and contains an α/β hydrolase fold common for lipases. Additionally, the NH_2_-terminal region shows high similarity to that of proline-β-naphthylamidase which is a member of the carboxylesterase family [60, 61]. Additional structural characteristics are an NH_2_-terminal-located ER signal peptide, a COOH-terminal ER-retention signal (HXEL), a predicted catalytic triad formed by Ser^221^, Glu^353 (354)^ and His^466 (468)^, and a hydrophobic stretch (AA 414–429) possibly involved in lipid binding [62–68]. In contrast to ATGL and HSL, TGH/Ces3 is mainly expressed in liver and to a low extent in WAT, kidney, cardiac muscle, and small intestine [66]. TGH/Ces3 mRNA level, protein expression, and concomitant hepatic microsomal esterase activity are decreased upon treatment with glucocorticoid analogs, mainly through destabilization of mRNA [69].

Intracellularly, TGH/Ces3 localizes to the ER, especially to areas surrounding cytosolic LDs [70]. TGH/Ces3 catalyzes the hydrolysis of long-, medium-, and preferentially short-chained TAGs [60] with so far unknown stereo/regioselectivity. Besides an unclear role in adipocytes, TGH/Ces3 is affirmed to be involved in the mobilization of hepatic TAG stores, required for the assembly of apoB100-lipoproteins, which has been deduced from the phenotype of mice carrying global disruption of *TGH/Ces3* gene [71].

#### DDHD domain containing 2 hydrolyzes TAGs in the brain

DDHD domain containing 2 (DDHD2), formerly known as KIAA0725p, is one of three sequence-related serine hydrolases which have been annotated as *sn*-1 specific phospholipases [72]. Mutations in DDHD2 are causative for the genetic disorder known as complex hereditary spastic paraplegia and cause intellectual disability, lower limb spasticity, and weakness.

The ~80 kDa large protein DDHD2 is ubiquitously expressed and exhibits highest expression levels in brain and adipose tissues. Within the cell, DDHD2 localizes to the Golgi network and exhibits phospholipase A1 (PLA1) activity against various PL substrates, including phosphatidic acid (PA), phosphatidylserine (PS), phosphatidylcholine (PC), and phosphatidylethanolamine (PE) [72]. However, a recent study identifies DDHD2 as intracellular TAG lipase involved in neutral lipid metabolism of the central nervous system [73]. DDHD2-deficient mice show significantly elevated TAG levels in brain, especially neurons, but not in other peripheral tissues. Similar, TAG accumulation pattern was observed using a selective DDHD2 inhibitor. Unexpectedly, no changes in the amount or composition of brain PLs were detected in DDHD2-deficient mice [73]. The stereo/region-selectivity of DDHD2 has not been investigated. However, since DDHD2 exhibits PLA1 activity, in addition to its TAG hydrolase activity, DDHD2 may hydrolyze TAG at the *sn*-1 position, generating *sn*-2,3 DAG.

#### Other intracellular triglyceride lipases

Several members of the PNPLA protein family, like adiponutrin (PNPLA3), GS2 (genes sequence 2, also annotated as PNPLA4), GS2-like (PNPLA5) as well as triacylglycerol hydrolase 2/carboxylesterase ML1 (TGH2), have been reported to possess TAG hydrolase activity in vitro and thus may be involved in cellular TAG catabolism.

TGH2 mRNA is highly expressed in WAT, BAT, and liver, and mRNA expression in adipocytes is induced upon differentiation [74]. A substantial amount of TGH2 protein localizes to LDs but TGH2 is also found in cytoplasmic and microsomal fractions. TGH2 shares around 70 % identity to murine TGH/Ces3 and contains a catalytic triad (Ser^221^, Glu^353^, His^466^ numbered for the murine protein), a hydrophobic stretch (414–429 AA), possibly involved in lipid binding, as well as a potential COOH-terminal ER retrieval sequence (HXEL) [74]. TGH2 exhibits hydrolytic activity for *para*-nitrophenyl butyrate, TAGs, and MAGs but no activity for DAGs, CEs, or PLs [74]. Analyses of FA- and glycerol release of isoproterenol-stimulated 3T3–L1 cells, infected with an adenovirus expressing TGH2, suggested a minor contribution (~20 %) of TGH2 to cellular lipolysis [74]. Besides the discovery that TGH2 prefers short-chained TAGs as substrates, nothing is known about the stereo-/regioselectivity of this enzyme.

Human *GS2* gene which has homologues in several mammalian genomes, except the mouse, is expressed in several tissues including WAT, skeletal muscle, cardiac muscle, liver, and skin (keratinocytes) [9, 14, 15, 75]. GS2 exhibits high homology to ATGL and contains an NH_2_-terminal-located catalytic dyad, including the canonical GXSXG lipase motif [14]. A variety of substrates is described for human GS2, including TAGs [14, 15], REs [14, 76], and PLs. Additionally, also a neutral lipid transacylase activity has been reported [8, 14]. Interestingly, the GS2 orthologue in rat displays lower RE hydrolase activity as well as differences in TAG hydrolase activity [14]. Whereas nothing is known about the FA preference, human GS2 is reported to hydrolyze TAGs at *sn*-1/(3) and *sn*-2 position, generating *rac*-1,3 and *sn*-1,2/(2,3) DAGs [14]. These products are not observable for rat GS2 which generates only *sn*-1,2/(2,3) DAGs. However, the physiological role of GS2 is unclear since no knockout model has been described and knockout mice cannot be generated (the mouse genome lacks the GS2 gene).

In contrast to GS2, GS2-like (also annotated as PNPLA5) mRNA is expressed to a low level in both mouse and human WAT, BAT, brain, and lung [9, 15]. The murine mRNA is upregulated during adipocyte differentiation and highly induced in liver samples of leptin-deficient mice [15]. In contrast, mRNA expression is markedly decreased during fasting [15]. Hydrolytic activity of GS2-like emerges to be enigmatic since non-tagged GS2-like protein exhibits no RE or TAG hydrolase activity in vitro [14] but lowers TAG accumulation when expressed in cells [15]. The stereo-/regioselectivity and the physiological relevance of GS2-like are unknown.

Adiponutrin (also annotated as PNPLA3) was first described as adipose tissue-specific transcript strongly regulated by the nutritional state [77]. Within the PNPLA family, adiponutrin shares highest sequence homology with ATGL. Adiponutrin, like all PNPLA proteins, contains a canonical serine lipase motif (GXSXG) within a catalytic dyad [10]. Adiponutrin is highly expressed in WAT, skin, and liver, and localizes to intracellular membranes and LDs [78, 79]. mRNA levels are highly increased by insulin or during adipocyte differentiation and are strongly decreased upon fasting (which is the opposite to ATGL mRNA expression) [7–9, 15, 77, 80]. Human adiponutrin exhibits TAG, DAG, MAG, and RE hydrolase as well as transacylase and PLA2 activity in vitro [8, 10, 15, 81]. Adiponutrin preferentially hydrolyzes TAG species containing long-chain unsaturated FAs (C18:1) thereby generating *sn*-1,2/2,3 DAGs (DAG species were not analyzed) [82]. However, overexpression or knock down of adiponutrin in mice did not affect hepatic TAG levels [83–85]. Notably, a human adiponutrin mutant (I148M) has been found to be strongly associated with nonalcoholic fatty liver disease and overexpression of the I148M mutant or a I148M knock-in in mice causes steatosis [84, 86]. In contrast to the reported hydrolytic activities, a recent study found that human and murine adiponutrin act as nutritionally regulated acyl-CoA-dependent lysophosphatidic acid acyltransferase and that this activity is elevated in the I148M mutant proteins [87]. Since decreased TAG hydrolytic activity as well as increased lysophosphatidic acid acyltransferase activity of the I148M mutant could explain the development of liver steatosis in humans, further studies are needed to clarify the physiological role of this protein.

### Phospholipase C generates *sn*-1,2 DAGs at the plasma membrane

Phospholipases specifically hydrolyze PLs at different chemical positions. The four major classes of phospholipases are distinguished by the type of catalyzed reaction. PLA1 and PLA2 catalyze the hydrolytic cleavage of the acyl chains at respective *sn*-1 or *sn*-2 position. In contrast, phospholipase C (PLC) and D (PLD) cleave phospho-glycerol and phospho-headgroup esters, respectively. Hence, only PLCs contribute directly to the intracellular formation of DAG from glycerophospholipids.

PLC activities have been grouped according to their substrate preference into phosphatidylcholine- and phosphatidylinositol-specific PLCs. Phosphatidylcholine-specific PLC (PC-PLC) are described in many organisms including mammals, in which the number of PC-PLC isoforms depends on cell type and species [88]. Localization and activity studies in murine fibroblasts as well as human lymphocytes showed PC-PLC expression and activity at perinuclear areas and upon mitogen-mediated cell receptor stimulation a translocation to the outer leaflet of the plasma membrane [89]. The fact that the gene sequence of mammalian PC-PLC has not been identified so far displays a critical hindrance since overexpression or deletion studies of mammalian PC-PLC cannot be performed. Thus, relatively little is known about the physiological role of mammalian PC-PLCs.

So far, thirteen phosphatidylinositol-specific PLC (PI-PLC) isozymes have been identified and assigned to six subclasses, namely β1–4, γ1–2, δ1, δ3–4, ε, ζ, and η1–2 [90–93]. Virtually all PI-PLC isozymes are highly expressed in different brain regions and only a few (β3, δ1, δ3, δ4, ε) are distributed among other peripheral tissues like liver, skeletal muscle or cardiac muscle [94]. Intracellularly, the soluble PI-PLC proteins are localized mainly in the cytoplasm. Upon cell activation PI-PLCs translocate to the plasma membrane and develop catalytic activity [94]. The PI-PLC-dependent hydrolysis of phosphatidylinositol 4,5-bisphosphate (PIP2), a plasma membrane-associated PL, describes a key event during regulation of a variety of cellular functions. By producing two intracellular messengers, namely *sn*-1,2 DAG and inositol 1,4,5-triphosphate (IP_3_), this reaction mediates activation of protein kinase C (PKC) as well as intracellular Ca^2+^ release, respectively [94]. Due to the *sn*-3 position of the phosphate residue of PLs, PI-PLC generates exclusively *sn*-1,2 DAGs [23].

### Sphingomyelin synthase generates *sn*-1,2 DAGs at the ER and the plasma membrane

Sphingomyelin synthases (SMSs) catalyze the transfer of the phosphorylcholine residue from PC to a ceramide backbone thereby generating sphingomyelin (SM) and *sn*-1,2 DAG [95, 96]. So far, two mammalian enzymes, SMS1 and SMS2 have been described [97, 98]. SMS1 localizes exclusively to the luminal side of the *trans*-Golgi whereas SMS2 additionally localizes to the extracellular leaflet of the plasma membrane [97, 99]. DAGs, produced by SMS1/2 at the *trans*-Golgi, may trigger the translocation of protein kinase D (PKD) which catalyzes the formation of secretory vesicles [100, 101]. In contrast, DAGs generated at the plasma membrane are most likely utilized by SMS2 to restock SM levels which are locally reduced by sphingomyelinases [102]. SMS-related protein (SMSr) was recently identified as third member of the SMS clan. SMSr localizes to the ER and converts ceramides, generated at the ER, to ceramide-phosphoethanolamine (CPE) using PE as donor [97, 103]. However, the CPE synthesis rate is very low as compared to those of SM synthesis. Thus, SMSr is supposed to regulate ceramide concentrations at the ER [103]. Substrate specificity studies including all three enzymes showed that SMS1 and SMSr are monofunctional SM and CPE synthases, respectively, whereas SMS2 is a bifunctional enzyme, able to catalyze both, SM and CPE synthesis [104]. All three enzymes are ubiquitously expressed in mammals and regulators of SM and ceramide homeostasis. Although the SMS family members exhibit a diverse substrate specificity, the fact that these enzymes use PLs as donors, suggests that the resulting DAG intermediates are *sn*-1,2 isomers.

### Synthesis of DAG de novo or by MGAT reaction

In addition to the catabolic generation of DAGs, two pathways contribute to the anabolic generation of DAGs. In those pathways DAG arises as intermediates of the de novo biosynthesis of TAGs and PLs. The G3P pathway is the major pathway of TAG synthesis in most tissues, predominantly in liver and WAT. The G3P-pathway involves the consecutive acylation of G3P, catalyzed by acyl-CoA-dependent glycerol-3-phosphate acyltransferase (GPAT) and acyl-CoA acylglycerol-3-phosphate acyltransferase (AGPAT). The product of these reactions is PA which is dephosphorylated to DAG by PA phosphatase (PAPase, lipins) [105–107]. Since G3P is utilized in this biosynthesis, these DAG isomers have exclusively the *sn*-1,2 conformation [108, 109]. In the other so-called MAG-pathway, *sn*-2 MAGs and long-chain fatty acyl-CoAs are esterified to *sn*-1,2 DAG by monoacylglycerol-acyltransferase (MGAT) [110]. This pathway plays a predominant role in enterocytes upon feeding and is also involved in the storage of TAGs in adipocytes [111, 112].

#### *sn*-1,2 DAG is generated in the G3P pathway at the ER

More than 50 years ago, it was discovered that the liver exhibits enzymatic activity that generates PA from glycerol and that DAG is a precursor for both PL and TAG biosynthesis. In fact, in the late 1950s, Kennedy and coworkers described an enzymatic activity that dephosphorylates PA to form DAG in vitro [113]. This finding completed the enzymatic sequence of TAG and PL synthesis from glycerol and is since then known as the Kennedy pathway [114, 115].

In mammals, PA dephosphorylation (3-*sn*-phosphatidate phosphatase/phosphohydrolase, PAP/lipin) is catalyzed by three recently identified members of the lipin family, namely lipin1, 2 and 3. Among these, lipin1 is the most extensively studied. It is highly expressed in tissues with high rates of lipid flux, like cardiac muscle, WAT, and skeletal muscle [116, 117]. In WAT and skeletal muscle, lipin1 accounts for the entire PAP activity, whereas the other two members contribute essentially to total PA dephosphorylation of liver, brain, and placenta [117, 118]. Early studies revealed that lipins locate within the cytoplasm and translocate rapidly to ER membranes upon elevated levels of intracellular FAs [119]. Loss-of-function mutations within lipin1 cause dramatic metabolic impairments, like hypertriglyceridemia and severe hepatic steatosis, as observed in fatty liver dystrophic mice [116]. The opposite effect is observed in transgenic mice overexpressing lipin1 in adipocytes. These mice exhibit increased amounts of TAG which fits to lipin1-dependent generation of DAGs as precursor for TAGs [120]. Since G3P and thus also PA are phosphorylated at *sn*-3 position of the glycerol backbone, their dephosphorylation generates exclusively *sn*-1,2 DAGs.

#### The MGAT reaction generates *sn*-1,2/2,3 DAGs at the ER in the digestive tract

MGAT enzymes catalyze the esterification of MAGs, which constitutes the first step in TAG synthesis following dietary absorption by enterocytes. So far, three enzymes are known to possess MGAT activity, MGAT1, -2, and -3. All three isoforms are located at the ER [121–125]. Besides similar intracellular localization, MGAT isoforms differ in their tissue-specific expression pattern and in their specific catalytic activities. In contrast to MGAT1 which is mainly expressed in stomach, adipose tissues, and kidney, MGAT2 and MGAT3 exhibit highest expression in small intestine [121–123, 125, 126]. MGAT3 is found exclusively in higher mammals and exhibits significantly higher specific DAG-acyltransferase activity as compared to MGAT1 and MGAT2. This indicates that MGAT3 functions as TAG synthase [127]. Furthermore, MGAT3 prefers *sn*-2 MAG as acyl acceptor and activated palmitic acid (C16:0) and C18:1 as acyl donors [123]. Thus, generated DAG species exhibit either *sn*-1,2 or *sn*-2,3 isomerism. The prior-mentioned lipids, *sn*-2 MAG, C16:0, and C18:1, are the major hydrolytic products of pancreatic triglyceride lipase/colipase (PTL)-dependent TAG hydrolysis during intestinal digestion [128, 129]. Hence, MGAT3 is thought to be main enzyme involved in the re-esterification of dietary absorbed fat within the small intestine.

MGAT2 was found to exhibit little or no selectivity concerning chain-length or level of saturation of activated FAs (FA-CoAs) [121]. Furthermore, incubation of MGAT2 with *rac*-1/3 MAGs results in the generation of *rac*-1,3 and *rac*-1,2/2,3 DAGs, implicating that all positions of the glycerol backbone can be esterified by MGAT2 [121]. In contrast, MGAT1 displays a stricter selectivity for glycerol position as well as for utilized FA-CoA species. MGAT1 shows high activity when incubated with long-chain unsaturated FA-CoAs, the highest with activated arachidonic acid (C20:4) [122]. Incubation with either *sn*-1 or *sn*-3 MAGs leads to the generation of *rac*-1,3 DAGs indicating that MGAT1 preferentially esterifies *sn*-1 or *sn*-3 position [122]. In line with these findings, incubation of *sn*-2 MAGs results exclusively in the generation of the *rac*-1,2/2,3 DAGs [122].

### Extracellular formation of DAGs

Diacylglycerols are also intermediates of the extracellular lipid metabolism. DAGs are generated in the process of food digestion as a result of TAG hydrolysis by specific lipases. Additionally, DAGs are generated in the circulation by the hydrolysis of lipoprotein-associated TAGs, by the action of lipoprotein lipase (LPL) and hepatic lipase (HL). Interestingly, all stereochemically characterized TAG lipases involved in lipid digestion, such as lingual lipase (LL), PTL, and gastric lipase (GL) exhibit preference for *sn*-1 or *sn*-3 position of TAGs. The specificity of pancreatic lipase-related proteins 1 and 2 (PLRP1/2) has so far not been determined. Furthermore, only PLRP2 but not PLRP1 is catalytically active against TAG [130–133]. PTL, GL, and LL hydrolyze TAG specifically at the *sn*-3 position but also hydrolyze DAGs. Thus, they break down TAGs into FAs and *sn*-2 MAGs [129, 134–137]. An example for a positional unspecific lipase is bile salt-stimulated lipase (BSSL, =carboxyl ester lipase, CEL) which hydrolyzes all positions of TAGs, generating free glycerol and FAs [138]. In contrast to the intestinal lipases, LPL and HL which deplete circulating lipoproteins from TAGs, exhibit *sn*-1 specificity for TAG and also hydrolyze DAGs at the *sn*-2 and *sn*-3 position, generating either *sn*-3 or *sn*-2 MAGs, respectively [134, 135, 139, 140]. Since the majority of these lipases not only generates but also accepts DAG as further substrate, the short half-life of extracellular DAGs makes them unlikely to be involved in intracellular signaling events. However, at least in the intestine the absorbed *sn*-2 MAG has signaling potential, since *sn*-2 arachidonoyl glycerol (2-AG) is an endogenous ligand of the cannabinoid receptors 1 and 2 (CB1/2) [141, 142]. 2-AG is known to retrogradely bind and activate CB1/2. Furthermore, recent evidence has shown that in the intestinum, 2-AG inhibits gut motility and propulsion [143–145].

## Intracellular metabolization of DAGs

**Fig. 4:**
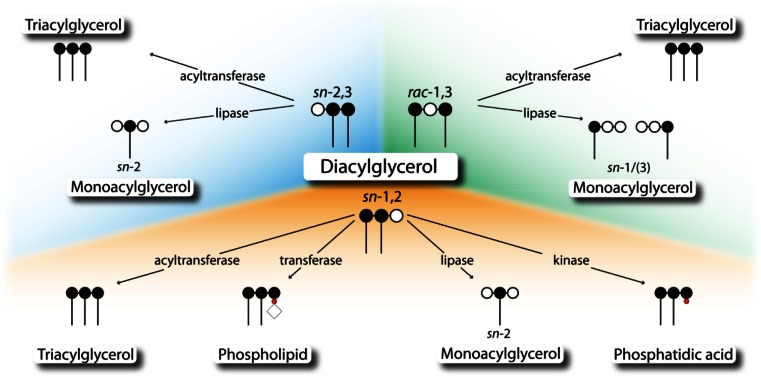
Intracellular enzyme classes involved in diacylglycerol utilization. Different isoforms of diacylglycerol display substrates for several enzyme classes, including transferases, kinases, and lipases. Carbon atoms of the glycerol backbone are depicted as *filled* (esterified) or *open* (unesterified) *circles*. Phosphate group, phosphate head group, and fatty acids are depicted as *red circle*, *open rhomb*, and *dash*, respectively

The intracellular metabolization of DAGs involves different enzymes, which in part exhibit selectivity for specific DAG isoforms. Thus, isomerism of DAG may influence (1) its degradation by lipases which leads to different regiomers of MAG species, (2) its re-esterification to TAG by DAG-specific acyltransferases, (3) its conversion to PC by CDP-choline:1,2-diacylglycerol choline phosphotransferases (CPTs), and (4) its phosphorylation by diacylglycerol kinases (DGKs) (Fig. 4).

### Diacylglycerol acyltransferases are distinct in intracellular localization and enzymatic preference

The acylation of DAG is the final step of TAG synthesis. This esterification reaction uses FA-CoA as acyl donor and DAG as acyl acceptor and is catalyzed by enzymes called diacylglycerol-*O*-acyltransferases (DGATs). So far, two mammalian DGAT enzymes have been identified, named DGAT1 and DGAT2.

DGAT1 was initially identified due to its high sequence similarity with acyl-CoA:cholesterol acyltransferase (ACAT) enzymes [146]. DGAT1 belongs to the large family of membrane-bound *O*-acyltransferases whose members catalyze the transfer of FAs onto thiol or hydroxyl groups of either lipid or protein acceptors [147]. DGAT1 mRNA is highly expressed in the small intestine, adipose tissues, skeletal muscle, cardiac muscle, skin, spleen, and testis, where it localizes strictly to ER membranes [127, 148, 149]. DGAT1 contains three transmembrane-spanning domains and an active site within the COOH-terminal region, facing the ER lumen [150]. Mutagenesis of a highly conserved histidine residue (His-426) located within the COOH-terminal region and supposed to be part of the active site abolishes the ability of DGAT1 to synthesize TAG in vitro [150]. The NH_2_-terminal region is located in the cytoplasm, facilitates the formation of tetramers, and binds long-chain FA-CoAs [150, 151]. Noteworthy, a C-terminal deletion of DGAT1 is inactive and does not act as a dominant negative protein [152]. The fact that microsomal preparations exhibit an “overt” (cytoplasmic) and a “latent” (luminal) DGAT activity [153], both influenced by pharmacological DGAT1 inhibition in vitro or genetically deletion of DGAT1 [154], questions whether the active site of DGAT1 is entirely oriented to the lumenal side of the ER membrane. In addition to the synthesis of TAG, DGAT1 catalyzes a diversity of different acyltransferase reactions, including MGAT, wax monoester synthase, and retinol acyltransferase [155].

DGAT2 shares little similarity with DGAT1. It belongs to a seven-member family including above-mentioned MGAT1, -2, and -3 [124, 156]. All members of this family contain the highly conserved amino acid sequence HPHG, which in case of DGAT2 is thought to be part of the active site [124, 147, 157]. Additionally, DGAT2 contains a FLXLXXXn consensus sequence which functions as a neutral lipid-binding domain, also found in other neutral lipid metabolizing proteins, such as plasma cholesteryl ester transfer protein, HSL, or TGH/Ces3 [62, 157, 158]. Mutation studies on DGAT2 demonstrated that this neutral lipid-binding domain of DGAT2 is responsible for DAG binding, since DGAT2 variants with mutations in this region exhibit reduced activities [157]. Expression of DGAT2 mRNA is highest in liver, adipose tissues, mammary glands, testis, peripheral leukocytes, and cardiac muscle [124]. In cultured cells DGAT2 localizes to the ER. Upon supplementation of cells with FAs, known to induce TAG synthesis, DGAT2 also localizes to mitochondria-associated membranes (ER domains tightly interacting with mitochondria) and to LDs [149, 159]. Unlike DGAT1, DGAT2 contains two ER-spanning domains and both, the COOH- as well as NH_2_-terminal domain, face the cytoplasm [157]. Hence, DGAT2 is in part responsible for the “overt” DGAT activity detectable in cells. Additionally, the C-terminus of DGAT2 is supposed to be involved in directing the enzyme to LDs, since DGAT2 with a truncated or mutated C-terminus fails to co-localize with LDs [160]. These different orientations of the active site of DGAT enzymes suggest a spatial separation of DGAT1 and DGAT2-dependent TAG synthesis. Furthermore, and in contrast to DGAT1, DGAT2 does not exhibit activities towards substrates other than DAGs.

Both DGAT enzymes are involved in the intracellular TAG synthesis. Overexpression of either DGAT1 or DGAT2 in mammalian cells leads to increased DGAT activity of corresponding cell lysates [161]. Interestingly, specific enzymatic activity of DGAT1 is higher than that of DGAT2. On the contrary, cells expressing DGAT2 accumulate more TAG, contain bigger LDs, and incorporate more glycerol into the TAG moiety as compared to that of DGAT1 expressing cells [161], questioning the significance of in vitro activity data for the prediction of physiological relevance.

DGAT1 and DGAT2 differ in their acyl donor and acyl acceptor preference. Studies on murine DGAT1 have revealed preference for monounsaturated acyl donors, like C18:1-CoA, over saturated FA-CoAs, like C16:0-CoA [124, 157]; yet, another study on human DGAT1 selectivity found equal preferences for both, C16:0-CoA and C18:1-CoA [162], questioning acyl donor preference of DGAT1. Intestinal human DGAT1 prefers *sn*-2 MAGs over *sn*-3 MAGs as substrates for the synthesis of *sn*-1,2 DAGs [162]. For DGAT2 no preferences for degree of saturation of FA-CoAs but a preference for medium-chained FA-CoAs (C12:0) as acyl donor and short- and medium-chained DAG species (e.g., C6:0, C8:0, C12:0) as acyl acceptor has been reported [124, 156]. Furthermore, DGAT enzymes differ in regard to the isomerism of DAGs as acyl acceptor molecules. DGAT1 utilizes *sn*-1,2/2,3 DAGs more efficiently than *rac*-1,3 DAGs. In contrast, DGAT2 exhibits the opposite acyl acceptor preference and prefers *rac*-1,3 DAGs [23].

An additional difference between DGAT enzymes is the sensitivity against magnesium which was demonstrated by in vitro experiments. High concentrations (>50 mM) are described to entirely suppress DGAT2 activity whereas DGAT1 activity is much less affected [124].

The differences in subcellular localization as well as enzymatic properties of DGATs suggest different intracellular functions. The observation that only DGAT2 localizes to LDs together with the preference for *rac*-1,3 DAGs suggests a coordinated action of DGAT2 and ATGL on LDs to facilitate a TAG hydrolysis/re-esterification cycle (futile cycle), potentially remodeling *sn*-2 FA ester species thereby replacing long-chain unsaturated with medium-chain fatty acids. Furthermore, the different intracellular localization of DGAT1/2 suggests that different DAG pools exist for DGATs. The one at LDs is only accessible for DGAT2. The other, at ER membranes, which is mostly supplied by DAG species (*sn*-1,2 DAGs) derived from the de novo glycero(phospho)lipid synthesis, is accessible for both DGAT1 and DGAT2. This spatial and stereochemical separation of different DAG pools as well as of executing enzymes could be one explanation for the different phenotype of mice carrying global deletions of DGAT1 or DGAT2. The phenotypes of DGAT-deficient mice clearly demonstrate that these enzymes cannot functionally compensate for each other [161, 163]. DGAT1 knockout mice exhibit a moderate phenotype characterized by reduced TAG levels in several tissues (e.g., adipose tissues, skeletal muscle, liver) and resistance to diet-induced obesity [163]. In contrast, DGAT2 knockout mice die within the first day of life most likely as consequence of a severe skin defect and severe lipopenia [161]. The exact reason for these drastic differences of DGAT knockout mice is unknown. Recently, distinct functions of DGAT1 and DGAT2 in hepatic TAG synthesis have been reported. DGAT2 is described to mediate esterification of newly synthesized FAs, whereas DGAT1 catalyzes the synthesis of TAGs via utilization of exogenously supplied FAs [164, 165]. Whether this finding holds true for other tissues needs to be tested.

### Diacylglycerol lipases hydrolyze *sn*-1,2 DAGs at the plasma membrane

HSL is well known to be rate limiting for DAG hydrolysis [48, 51]. Yet, there are additional intracellular lipases which possess specific hydrolytic activities for DAGs, generating MAGs and FAs. In humans, two *sn*-1-specific DAG lipases have been identified, named DAG lipase α and β (DAGLα and β) [166]. Both enzymes share ~30 % homology and comprise four predicted transmembrane-spanning domains as well as a serine lipase motif [166].

DAGLα is highly expressed in brain and pancreas and to a lower extent in macrophages and adipose tissues, while DAGLβ expression is highest in bone marrow, lymph nodes, spleen, and liver [166, 167]. Enzymatic characterization revealed that both enzymes hydrolyze the *sn*-1,2 DAG isomer (*sn*-2,3/*rac*-1,3 were not tested), exhibiting three- to eightfold higher activity for the *sn*-1 over the *sn*-2 position of DAGs [166]. Furthermore, DAGLβ prefers DAG species, containing linoleic acid (C18:2) > C18:1 > C20:4 > stearic acid (C18:0) at *sn*-2 position, whereas DAGLα shows equal activity against these DAG species [166].

Intracellularly, DAGLα localizes to the plasma membrane and hydrolyzes *sn*-1,2 DAGs generated by PLC-dependent hydrolysis of phosphatidylinositides [168]. In contrast, DAGLβ also localizes to the LDs [168]. DAGL-mediated breakdown of *sn*-1,2 DAGs at the plasma membrane results in the formation of *sn*-2 MAGs. Since PLs, the precursors of PLC-derived DAGs, exhibit a high abundance of C20:4 esterified at the *sn*-2 position of the glycerol backbone, the released MAG species is frequently 2-AG [169, 170]. As already mentioned above, 2-AG is the most abundant endocannabinoid in tissues and acts as ligand for cannabinoid receptors CB1 and CB2 [171]. In fact, the role of DAGLα and DAGLβ in the biosynthesis of 2-AG is evident from the phenotype of DAGLα or DAGLβ-deficient mice [167]. In line with the tissue expression patterns, DAGLα knockout mice displayed 80 % reduction in 2-AG levels in the brain, whereas DAGLβ knockout mice showed 90 % reductions in 2-AG levels in the liver [167].

The role of DAGL enzymes in the hydrolysis of plasma membrane-derived *sn*-1,2 DAG species is well established. To date, it is questionable whether *sn*-1-specific DAGL enzymes are also involved in the degradation of DAG species of other sources such as *rac*-1,3 DAGs which originate from lipolysis-driven TAG breakdown at the LDs or *sn*-1,2 DAGs which also derive from de novo synthesis at the ER membrane.

### Diacylglycerol kinases are ubiquitously expressed and phosphorylate *sn*-1,2 DAG

Diacylglycerol kinases (DGKs) catalyze the formation of PA by phosphorylation of the free hydroxyl (–OH) group of DAG. Together with PAPases/lipins, DGKs are crucially involved in the maintenance of intracellular DAG and PA levels. So far, ten DGK isozymes have been identified in mammals [172, 173]. All mammalian DGK isozymes share two common structural features, a cysteine-rich C1 domain which is responsible for DAG binding and potentially involved in protein–protein interaction and a catalytic domain, responsible for enzymatic activity [174]. Virtually every tissue expresses at least one member of the DGK family. However, numerous tissues express several different DGK isozymes, e.g., all ten DGK isozymes can be found in brain extracts [175]. DGKs localize to multiple cellular compartments, including the nucleus, the plasma membrane, the cytoskeleton, the Golgi network, and the ER [176, 177]. Little is known about the specific functions of different DGK enzymes with regard to their organelle distribution. Whereas some isoforms translocate to the plasma membrane upon activating stimuli (most likely to funnel PLC-derived DAGs to PA), others locate inside the nucleus, presumably regulating nuclear DAG levels [174].

DGKs are selective for *sn*-1,2 DAGs. Most likely, this is due to their C1 domain which shares high homology to the *sn*-1,2 DAG-specific binding motifs C1A and C1B of PKC [178–180]. However, to date it is unclear whether DGK isoforms are also capable to phosphorylate *rac*-1,3 or *sn*-2,3 DAG isoforms. Further enzymatic properties are known for DGKε. DGKε is the shortest DGK isoform which exhibits the lowest molecular weight and localizes either to the plasma membrane or to the ER. DGKε exhibits specificity for *sn*-1,2 DAG species [181] containing C18:0 at the *sn*-1 position and C20:4 at the *sn*-2 position [182, 183] which is the major product of PLC-mediated phosphoinositide hydrolysis [169]. DGKε is thought to terminate 20:4-DAG signaling and to counterbalance PIP2 hydrolysis by specifically phosphorylating C20:4-containing DAGs to PA. In turn, generated PA would then be available for re-synthesis of PI and replenishment of PIP2 by consecutive phosphorylations to phosphatidylinositol 4-phosphate (PIP) and PIP2. This hypothesis is supported by the phenotype of mice carrying a global gene deletion DGKε which show downregulation of the 20:4-DAG to 20:4-PI lipid cycle [184].

### Diacylglycerol choline/ethanolamine phosphotransferases specifically utilize *sn*-1,2 DAGs at intracellular membranes

All tissues and cell types can synthesize PC via the CDP-choline or better known as “Kennedy” pathway [114, 115]. In an analog manner, PE can be formed via the CDP-ethanolamine pathway. The final step of both pathways, namely the direct conversion of DAGs to either PC or PE is catalyzed by CPT or CDP-ethanolamine:1,2-diacylglycerol ethanolaminephosphotransferase (EPT), respectively.

In humans, two proteins exhibit CPT activity. CPT1 acts as CDP-choline-specific enzyme while CEPT1 transfers both CDP-choline as well as CDP-ethanolamine [185, 186]. Both proteins are integral membrane proteins and localize to the Golgi network and the ER membrane, respectively [187]. Human CPT1 mRNA expression is highest in testis, colon, small intestine, cardiac muscle, and spleen, whereas CEPT1 is expressed to a similar degree in all tissues examined [185, 186]. Due to the high CPT activity in cells, none of the two enzymes is thought to be rate-limiting in PC synthesis [188]. Since no purified mammalian CPT enzyme is available, information on substrate specificity is only available from structure/function analyses of CPT1 of *S. cerevisiae*. The yeast orthologue prefers *sn*-1,2 DAGs (*sn*-2,3/*rac*-1,3 were not tested), with a FA preference in the order of dipalmitolein (diC16:1) > diC16:0 = C18:1/C16:0 > C16:0/C18:1 DAG as substrates [189]. Selectivity studies using microsomal fractions of *S. cerevisiae* constitutively expressing human CEPT1 suggest a diC16:1 > C16:0/docosahexaenoic acid (C22:6) > C16:0/C18:1 > diC18:1 DAG species preference for CEPT1 [187]. The third enzyme, ethanolamine phosphotransferase 1 (EPT1) selectively catalyzes the transfer of CDP-ethanolamine on *sn*-1,2 DAGs (*sn*-2,3/*rac*-1,3 were not tested), forming PE [190], and contributes only to 5 % to cellular PC synthesis [191, 192]. Furthermore, EPT1 of *S. cerevisiae* prefers *sn*-1,2 DAGs (*sn*-2,3/*rac*-1,3 were not tested) species, with diC18:1 > diC16:1 > C16:0/C18:1 as FA chains in vitro [189]. Apart from the enzymatic activity, little is known about EPT1 expression pattern or biochemical properties. Interestingly, incubation of rat hepatocytes with radiolabeled ethanolamine results in a high specific accumulation of radioactivity in C16:0/C22:6-PE, suggesting that in mammals EPT activity may exhibit preference for C16:0/C22:6-DAGs as substrates [193].

Taken together, CPT1, CEPT1, and EPT1 are involved in the synthesis of PLs, thereby consuming *sn*-1,2 DAGs.

## How DAG kicks into insulin signaling

Besides the capability of DAGs to negatively regulate transient receptor potential canonical (TRPC) cation channels [194, 195], dysregulations of DAG metabolism and concomitant DAG accumulation is supposed to adversely affect cellular signaling involved in the development of different disease states, like insulin resistance (IR). Intracellular accumulation of DAG is thought to be connected to altered insulin responsiveness since ectopic DAG accumulation positively correlates with disturbed insulin signaling. This adverse effect of DAGs is thought to derive from the action of the PKC family members which are known to play a crucial role in many signaling pathways which control, e.g., cellular differentiation, and cell growth. The PKC family comprises three different PKC subgroups, namely conventional (α, β1, β2 and γ; cPKC), novel (δ, ε, η and θ; nPKC) and atypical (ζ and λ/ι; aPKC) PKCs. cPKCs and nPKCs display lipid-sensitive isoforms and are usually activated by PLC-derived *sn*-1,2 DAGs, either Ca^2+^ dependent (cPKC) or independent (nPKC). The C1 domain of conventional and novel PKCs binds DAGs and the tumor promoter phorbol ester with high affinity [196–198]. Hence, the activity of cPKCs and nPKCs is highly influenced by intracellular levels of DAGs. Noteworthy, earlier studies showed that only the *sn*-1,2 DAG isoform has the ability to activate PKCs, the other isoforms, *rac*-1,3 and *sn*-2,3 are inactive [178–180]. Binding of cellular DAGs to members of the PKC family leads to their activation and translocation to the plasma membrane and subsequent phosphorylation of interacting proteins (such as insulin receptor substrate, IRS). It is assumed that a rise in cellular DAG levels may activate PKCs in an uncontrolled fashion.

So far, cellular effects driven by DAG accumulation have been mainly attributed to cPKCs and nPKCs. However, other “non-PKC” proteins also contain a high-affinity DAG/phorbol ester-binding C1 domain. To date a variety of DAG receptor proteins are described which belong to the families of PKD kinases, chimaerin Rac GTPase-activating proteins, Ras guanyl nucleotide-releasing proteins (RasGRPs), Munc13 scaffolding proteins, and former-mentioned DAG kinases [199–205]. Importantly, members of these families have been described to affect cellular processes like cell adhesion (chimaerins) [206], cell proliferation/transformation (RasGRPs) [207, 208], secretory vesicle priming (Munc13s) [209–212], and protein transport (PKDs) [213, 214]. Thus, biological effects driven by DAG binding to proteins of these families must be considered when cellular DAG signaling is investigated. Several reviews on the physiological role of non-PKC C1 domain-containing proteins have elaborated the current knowledge [199–205]. The emerging number of metabolic diseases as result of the first world life style brings DAG-dependent PKC signaling and related attenuation of insulin action in the focus of a multitude of present-day studies. Therefore, we focus in the following section on the PKC signaling axis in more detail.

**Fig. 5:**
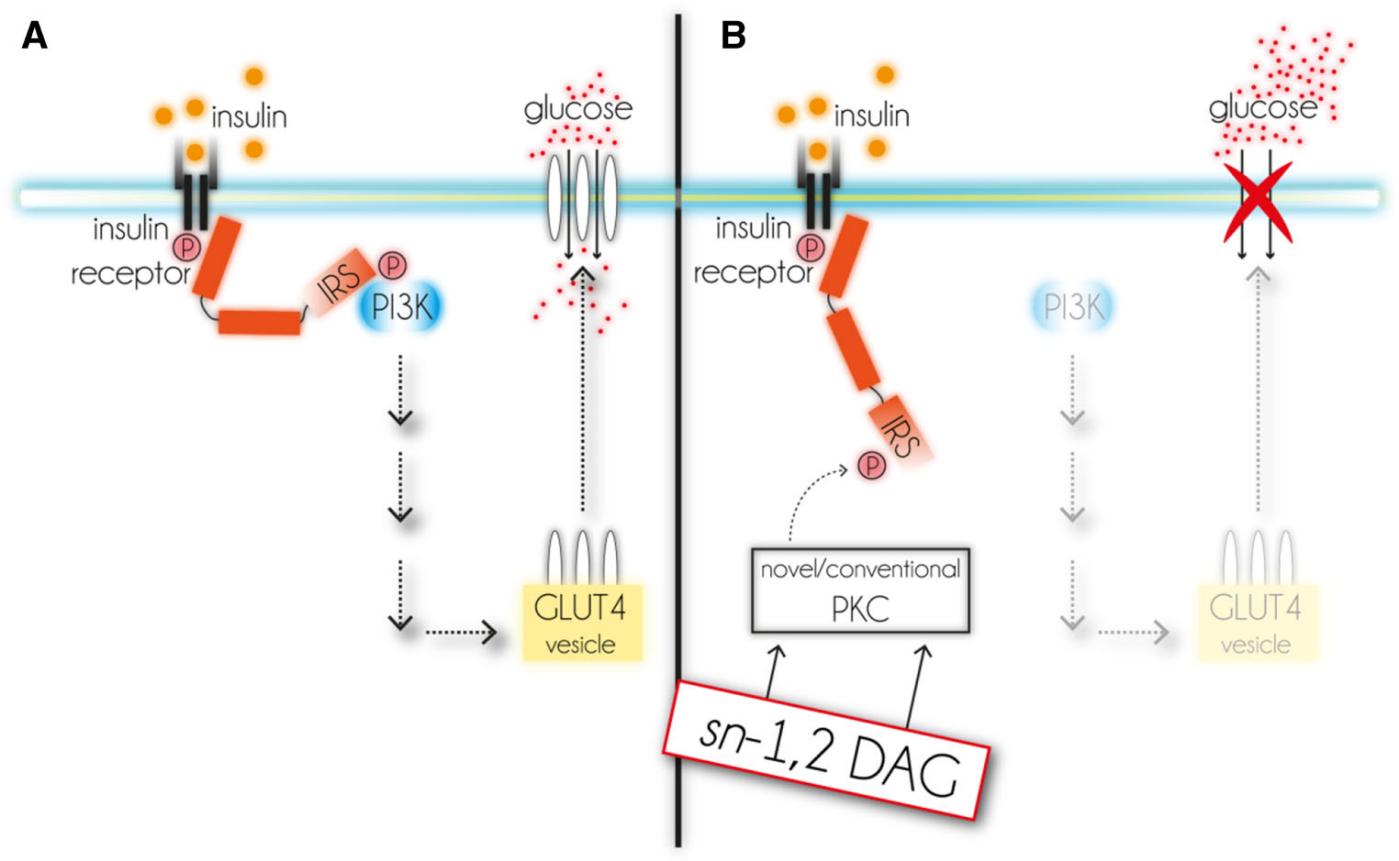
Intracellular pathway of insulin signaling and proposed impairment by DAG. **a** Insulin binds to insulin receptor that activates IRS. This leads to the activation of PI3K and further downstream signaling which finally triggers the translocation of GLUT4 to the plasma membrane and enables glucose uptake. **b** *sn*-1,2 DAG activates novel and conventional PKC isoforms which phosphorylate IRS. This event inhibits downstream effector signaling and GLUT4-dependent glucose uptake. *DAG* diacylglycerol, *GLUT4* glucose transporter 4, *IRS* insulin receptor substrate, *P* phosphorylation, *PI3K* phosphoinositide-3-kinase, *PKC* protein kinase C

Insulin signaling in myocytes and adipocytes is initiated by the binding of insulin to its receptor at the plasma membrane. Intracellularly, this activates a tyrosine kinase which consecutively phosphorylates one of the intracellular IRS 1-4 family members on several tyrosine residues [215, 216]. Activated IRSs serve as docking station for proteins like phosphatidylinositide-3-kinase (PI3K) and induce downstream effects which finally result in the translocation of glucose-transporter type 4 (GLUT4) to the plasma membrane [217]. Only plasma membrane-associated GLUT4 facilitates glucose uptake (Fig. 5a). Defects within this signaling cascade result in a loss of insulin sensitivity and consequently to IR of affected cells/tissues.

Of all PKC subgroups mainly nPKCs, more precisely PKCε and PKCθ, adversely affect insulin signaling [218, 219]. Up to now several mechanisms have been identified by which nPKCs impair insulin action. Recent studies showed that PKCθ can phosphorylate IRS1 at Ser^1101^ which blocks insulin-stimulated tyrosine phosphorylation [220] and activation of PI3K [218]. As a consequence, GLUT4 does not translocate to the plasma membrane and glucose is not taken up (Fig. 5b).

## Mouse models for deregulated DAG metabolism

Deregulation of DAG metabolism and concomitant DAG accumulation is thought to adversely affect cellular signaling and to be causally related to the development of various disease states, including IR. The phenotype of a number of genetic mouse models supports the hypothesis that increased DAG levels in insulin responsive tissues are causative for impaired insulin signaling. For example, overexpression of mitochondrial GPAT in mice leads to elevated levels of lysophosphatidic acid (LPA), DAG, and TAG in the liver and these mice develop hepatic IR in absence of a lipogenic diet or obesity [221]. Similarly, in obese Zucker rats (rats with non-functional leptin receptor) IR is associated with increased amounts of hepatic and muscle ceramide and DAG levels [222]. Another genetic mouse model arguing for DAG as mediator of IR are mice fed a high-ketogenic diet. These mice develop severe hepatic steatosis and severe hepatic IR which is associated with a 350 % increase in hepatic DAG content [223]. These mice exhibit elevated levels of activated PKCε and decreased insulin-mediated tyrosine phosphorylation of IRS2. In mice overexpressing DGAT2 specifically in the liver, hepatic TAG as well as DAG and ceramide levels are markedly increased [224, 225]. These mice were first reported to have normal hepatic insulin sensitivity [224] but were recently identified to exhibit enhanced PKCε activation, accompanied by severe hepatic IR [225]. Furthermore, an interrelation between cellular DAG levels, PKCε activation, and hepatic IR has been observed in numerous models and has been extensively reviewed elsewhere [226]. Interestingly, a recent study discovered that hepatic DAG content of cytoplasmic LDs is the best predictor of IR in obese, non-diabetic individuals [227]. In the same study, they observed distinct localization of PKCε at cytoplasmic LDs as well as enhanced activation of PKCε [227]. So far, this study is unique in connecting DAG turnover at the LD with PKC signaling.

The phenotype of a number of other genetic mouse models argues against a causative role of non-plasma membrane-derived DAGs in the development of IR. For example, HSL knockout mice accumulate large amounts of DAG in adipose and non-adipose tissues [48, 51] but generally they do not develop IR. Unfortunately, the issue of whether or not HSL-deficient mice develop IR is conflicting since depending on the genetic background, some HSL knockout strains show signs of impaired insulin signaling [228, 229] while others do not or show even increased insulin sensitivity [230, 231]. In contrast to mice, however, humans with mutated HSL develop severe diabetes which could be a result of ectopic lipid deposition in liver in combination with partial lipodystrophy [232]. Accumulation of *rac*-1,3 DAG, as determined in HSL-ko mice, is unlikely to be causative for the development of IR since *rac*-1,3 DAG isomers are known not to activate PKCs [23, 178–180].

Another mouse model with cellular DAG accumulation was generated by antisense oligonucleotides (ASO)-mediated silencing of CGI-58 expression. ASO injection into mice mainly targets the liver, adipose tissues, and kidney and does not lead to a global silencing of expression. ASO-CGI-58 mice on a high-fat diet regimen exhibit increased hepatic TAG, DAG, and ceramide levels. However, animals have been shown to exhibit improved insulin sensitivity and glucose tolerance [233]. The lack of adverse insulin signaling despite DAG accumulation in ASO-CGI-58-treated mice was explained by the sequestration of DAG molecules in LDs so that they were unable to interact with PKCs [234].

A recent study reported that overexpression of ATGL in cultured myotubes leads to increased DAG and ceramides levels which was found to be associated with impaired insulin signaling [235]. This suggests that deregulation of TAG metabolism might be linked to insulin signaling. Yet, mice globally lacking ATGL or mice overexpressing ATGL specifically in adipose tissue display also deregulated TAG metabolism, but show improved glucose tolerance [22] or are protected from IR [236], respectively, questioning a causal link between LD-associated TAG deregulation and insulin signaling.

In summary, several of the above-mentioned studies suggest a causal link between DAG, PKC signaling, and the development of IR. In this context, DAG isomerism as well as intracellular DAG compartmentation might be of crucial importance. As outlined above, a number of metabolic reactions degrade or consume DAGs. Yet, they are located at different cellular compartments and DAG isoforms involved in these reactions are stereochemically different. Earlier studies clearly demonstrated that the DAG-binding C1 domain of PKCs is highly specific for *sn*-1,2 DAGs. Other isoforms, like *sn*-2,3 or *rac*-1,3 DAGs, are unable to activate PKCs [178–180] and hence display no signaling properties. Due to the inability of DAG isoforms, other than *sn*-1,2 DAG, to activate PKCs it appears crucial to precisely determine DAG isomerism under normo-physiological and patho-physiological conditions.

## 3-Pool model of DAG metabolism, conclusions, and perspectives

DAG metabolism involves a battery of enzymes at spatially different cellular locations with distinct enzymatic properties and selectivities. For the sake of simplicity many studies in the past have ignored spatial and stereochemical issues and have correlated total cellular DAG levels with PKC activation as proof for causality between cellular DAG levels and DAG signaling. Future studies on DAG-producing or -consuming enzymes need to address these issues of stereoselectivity of DAG species and their cellular localization. This will help to further elaborate intracellular DAG metabolism and functions of different DAG isomers which is required to comprehend the metabolic implications of normo-physiological and pathological DAG metabolism and function. To date it is appreciated that DAGs exist in different stereo/regioisoforms and it is assumed that certain DAG isoforms exhibit specified intracellular functions at different cellular locations and processes. Furthermore, the amount of DAGs at different sites (e.g., intracellular membranes *vs* plasma membranes) depends on various stimuli or metabolic processes and underlies a remarkable spatiotemporal dynamic [237–239].

The stereochemistry of DAG isomers as well as the distinct localizations and activities of DAG-generating/consuming enzymes suggest a spatial separation of DAG pools which can be outlined in a “3-pool model” of intracellular DAG compartmentation [23]. This model describes that 3 distinct intracellular DAG pools exist at the ER/Golgi network, the LD, and the plasma membrane which differ to some extent in their DAG isomerism and are accessible for different sets of enzymes.

**Fig. 6:**
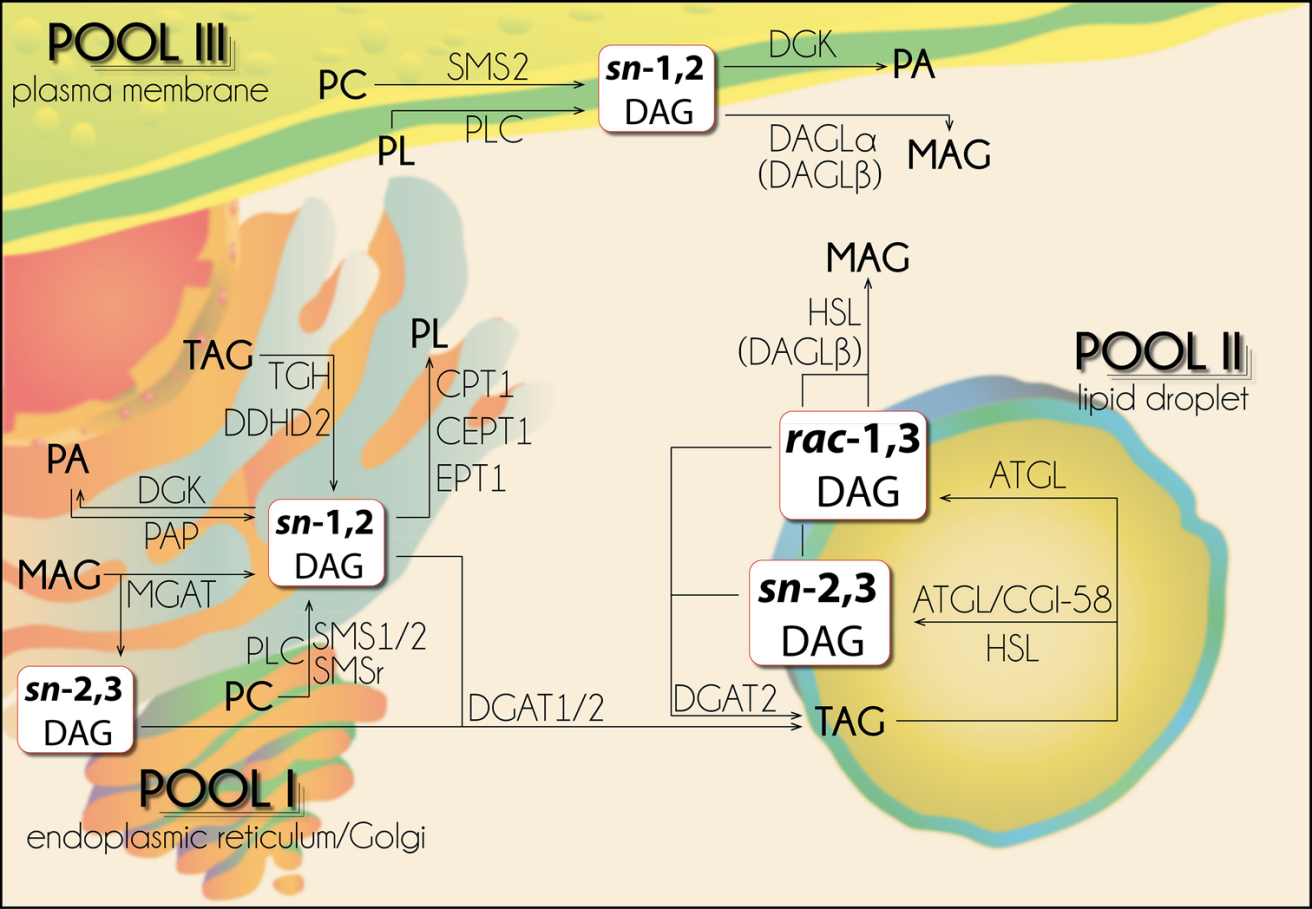
“3-Pool” compartmentation model of intracellular diacylglycerols. Intracellular diacylglycerols differ in their stereo/regio conformation, compartmentation, and generating/consuming enzymes. For detailed description see text. *ATGL* adipose triglyceride lipase, *CGI-58* comparative gene identification-58, *CEPT* CDP-ethanolamine/choline:1,2-diacylglycerol ethanolaminephosphotransferase CPT, CDP-choline:1,2-diacylglycerol ethanolaminephosphotransferase, *DAG* diacylglycerol, *DAGL* DAG lipase, *DGAT* DAG-O-acyltransferase, *DGK* DAG kinase, *EPT* CDP-ethanolamine:1,2-diacylglycerol ethanolaminephosphotransferase, *HSL* hormone-sensitive lipase, *MAG* monoacylglycerol, *MGAT* monoacylglycerol-*O*-acyltransferase, *PA* phosphatidic acid, *PAP* PA phosphohydrolase/lipin, *PC* phosphatidylcholine, *PL* phospholipid, *PLC* phospholipase C, *SMS(r)* sphingomyelin synthase (related), *TAG* triacylglycerol

“Pool I” is located at the ER/Golgi network and consists of *sn*-1,2 DAGs. This DAG pool is generated by de novo glycero(phospho)lipid synthesis either by PAPases/lipins or MGAT enzymes, as by-product of SM synthesis catalyzed by SMSs, or as product of TAG hydrolysis by lipases like TGH/Ces3 or DDHD2. Furthermore, *sn*-1,2 DAGs of this pool can be utilized by DGKs forming PA, DGAT enzymes generating TAG, and PL-generating enzymes like CEPT1, CPT1, and EPT1 (Fig. 6).

Lipolysis-derived DAGs represent “Pool II” which is located at cytoplasmic LDs. This pool comprises *rac*-1,3 and *sn*-2,3 DAGs and is fueled by the TAG hydrolytic activities of ATGL and HSL. The *rac*-1,3 DAG isomer is generated by TAG hydrolysis of ATGL. The *sn*-2,3 DAG isomer is generated by TAG hydrolysis of HSL and of ATGL, in addition to the *rac*-1,3 DAG isomer, when co-activated by CGI-58. DAGs of this pool can be re-esterified to TAGs exclusively by DGAT2 or further be catabolized to *sn*-2 MAGs by HSL(or DAGLβ)-mediated hydrolysis (Fig. 6).

“Pool III” is located at the plasma membrane and consists of *sn*-1,2 DAGs. These *sn*-1,2 DAGs are generated either by PLC-dependent breakdown of PLs or during SM production catalyzed by SMS2. DAGs of “Pool III” can serve as precursors for the synthesis of PA through DGK-catalyzed phosphorylation or can be hydrolyzed to *sn*-2 MAGs by membrane-associated DAGLα (or DAGLβ). So far, only *sn*-1,2 DAGs of “Pool III” are established to be involved in DAG signaling events, executed by nPKC and cPKC isoforms (Fig. 6).

The differences in stereo/regiochemistry of involved DAGs as well as the topological separation of these three DAG pools may provide explanations to previously puzzling data in regard to DAG-induced signaling, lipid metabolism, and metabolic disorders. Furthermore, the involvement of so far unidentified DAG-specific binding proteins and/or DAG isomerases could interconnect different intracellular sources of DAGs and could explain seemingly contradicting findings on DAG function and signaling potential. Following a multiplicity of studies focusing on the role of DAGs and in-depth reviews of DAG metabolism and functions [240–242], the future attention will further shift to the question which role is attributed to different DAG isomers as well as to different DAG species, in respect to their FA composition, in intracellular metabolism. Unraveling these issues will finally paint a more complete picture of the interrelation between lipid metabolism, intracellular signaling, and certain disease pathologies.

## Abbreviations

2-AG: 2-Arachidonoyl glycerol
AGPAT: Acyl-CoA acylglycerol-3-phosphate acyltransferase
ATGL: Adipose triglyceride lipase
BAT: Brown adipose tissue
BSSL/CEL: Bile salt-stimulated lipase/carboxyl ester lipase
CGI-58: Comparative gene identification-58
CE: Cholesteryl ester
CEPT: CDP-ethanolamine/choline:1,2-diacylglycerol ethanolaminephosphotransferase
CPT: CDP-choline:1,2-diacylglycerol ethanolaminephosphotransferase
DAG: Diacylglycerol
DAGL: DAG lipase
DGAT: DAG-*O*-acyltransferase
DGK: DAG kinase
EPT: CDP-ethanolamine:1,2-diacylglycerol ethanolaminephosphotransferase
ER: Endoplasmic reticulum
FA: Fatty acid
G3P: Glycerol-3-phosphate
GL: Gastric lipase
GLUT4: Glucose transporter 4
GPAT: Glycerol-3-phosphate acyltransferase
HSL: Hormone-sensitive lipase
IR: Insulin resistance
IRS: Insulin receptor substrate
LD: Lipid droplet
LL: Lingual lipase
LPA: Lysophosphatidic acid
LPL: Lipoprotein lipase
MAG: Monoacylglycerol
MGAT: Monoacylglycerol-*O*-acyltransferase
PA: Phosphatidic acid
PAP: PA phosphohydrolase/lipin
PC: Phosphatidylcholine
PDK: 3-Phosphoinositide-dependent protein kinase
PE: Phosphatidylethanolamine
PI: Phosphatidylinositol
PI3K: Phosphatidylinositide-3-kinase
PIP: Phosphatidylinositolphosphate
PIP2: Phosphatidylinositol 4,5-bisphosphate
PKB/Akt: Protein kinase B
PKC: Protein kinase C
PKD: Protein kinase D
PL: Phospholipid
PLA1(2): Phospholipase A1 (2)
PLC: Phospholipase C
PLD: Phospholipase D
PLRP: Pancreatic lipase-related protein
PNPLA: Patatin-like phospholipase domain containing
PS: Phosphatidylserine
PTL: Pancreatic triglyceride lipase/colipase
RE: Retinyl ester
SMS: Sphingomyelin synthase
SMSr: SMS-related protein
TGH/Ces3: Triacylglycerol hydrolase/carboxylesterase 3
TAG: Triacylglycerol
TRPC: Transient receptor potential canonical
WAT: White adipose tissue
